# Particulate matter promotes cancer metastasis through increased HBEGF expression in macrophages

**DOI:** 10.1038/s12276-022-00886-x

**Published:** 2022-11-09

**Authors:** Seung-Ho Park, Sung-Jin Yoon, Song Choi, Jaeeun Jung, Jun-Young Park, Young-Ho Park, Jinho Seo, Jungwoon Lee, Moo-Seung Lee, Seon-Jin Lee, Mi-Young Son, Young-Lai Cho, Jang-Seong Kim, Hyo Jin Lee, Jinyoung Jeong, Dae-Soo Kim, Young-Jun Park

**Affiliations:** 1 Environmental Disease Research Center, Korea Research Institute of Bioscience and Biotechnology (KRIBB), Daejeon, Republic of Korea; 2 Department of Medical Science, Chungnam National University School of Medicine, Daejeon, Republic of Korea; 3 Futuristic Animal Resource and Research Center, KRIBB, Ochang, Republic of Korea; 4 University of Science and Technology (UST), Daejeon, Republic of Korea; 5 Stem Cell Convergence Research Center, KRIBB, Daejeon, Republic of Korea; 6 Metabolic Regulation Research Center, KRIBB, Daejeon, Republic of Korea; 7 Biotherapeutics Translational Research Center, KRIBB, Daejeon, Republic of Korea; 8 Department of Internal Medicine, Cancer Research Institute and Infection Control Convergence Research Center, Chungnam National University College of Medicine, Daejeon, Republic of Korea

**Keywords:** Metastasis, Monocytes and macrophages

## Abstract

Although many cohort studies have reported that long-term exposure to particulate matter (PM) can cause lung cancer, the molecular mechanisms underlying the PM-induced increase in cancer metastasis remain unclear. To determine whether PM contributes to cancer metastasis, cancer cells were cultured with conditioned medium from PM-treated THP1 cells, and the migration ability of the treated cancer cells was assessed. The key molecules involved were identified using RNA-seq analysis. In addition, metastatic ability was analyzed in vivo by injection of cancer cells into the tail vein and intratracheal injection of PM into the lungs of C57BL/6 mice. We found that PM enhances the expression of heparin-binding EGF-like growth factor (HBEGF) in macrophages, which induces epithelial-to-mesenchymal transition (EMT) in cancer cells, thereby increasing metastasis. Macrophage stimulation by PM results in activation and subsequent nuclear translocation of the aryl hydrocarbon receptor and upregulation of HBEGF. Secreted HBEGF activates EGFR on the cancer cell surface to induce EMT, resulting in increased migration and invasion in vitro and increased metastasis in vivo. Therefore, our study reveals a critical PM-macrophage-cancer cell signaling axis mediating EMT and metastasis and provides an effective therapeutic approach for PM-induced malignancy.

## Cancer: Particulate matter pollutants promote progression

The inhalation of tiny particles known as particulate matter (PM) from polluted air induces an inflammatory response in the lungs that may contribute to tumor metastasis. PM contributes to a host of respiratory problems including lung cancer, and many of these toxic effects are thought to arise from chemical signals released by immune cells upon exposure to these pollutants. Seung-Ho Park at the Korea Research Institute of Bioscience and Biotechnology, Daejon, South Koread, and coworkers have determined that immune cells exposed to PM secrete HBEGF, member of the EGF family of growth factors, which causes cultured cancer cells to transition into a more invasive, metastatic state. They observed a similar process in mice injected with cancer cells, where exposure to PM resulted in elevated HBEGF production and increased numbers of metastatic tumors.

## Introduction

Air pollution caused by rapid industrialization and urbanization seriously threatens human health worldwide^1,2^. According to the 2019 Global Burden of Disease study, particulate matter (PM) ranks fifth among mortality risk factors, causing 292,500 deaths and 13 million disability-adjusted life years^1^. PM can deeply penetrate the respiratory system and is closely related to the incidence and exacerbation of human respiratory diseases, such as asthma, chronic obstructive pulmonary disease, and lung cancer^3^. A meta-analysis using data from 17 cohort analyses in nine European countries in 2013 showed that long-term exposure to PM is linked to lung cancer, especially lung adenocarcinoma. PM exposure has been reported to increase lung cancer incidence, even at concentrations below the existing European Union air quality limit values for PM10 (40 μg/m^3^) and PM2.5 (25 μg/m^3^)^4^. Furthermore, the International Agency for Research on Cancer has classified PM in outdoor air as a Group I carcinogen and confirmed that exposure to PM increases lung cancer risk^5^.

The respiratory system is the main route for the entry of PM into the lungs. PM larger than 10 μm is mostly trapped in the nasopharynx and throat and does not enter the lungs, while PM smaller than 10 μm is removed from the tracheobronchial tree through mucociliary clearance^6^. As PM smaller than 2.5 μm can enter the alveolar space, macrophages are recruited to the site of PM infiltration in response to cytokine and chemokine induction. The recruited macrophages can internalize PM through phagocytosis^7,8^ and secrete various proinflammatory cytokines in response to oxidative stress and local inflammation in the infiltrated area^9^. The increases in cytokine and chemokine levels can affect various stages of cancer metastasis^10^, while infiltration of alveolar macrophages and interstitial macrophages in the lungs enhances metastasis^11^.

Epithelial-to-mesenchymal transition (EMT) is a major alteration occurring in the earliest stages of cancer metastasis^12–14^. Transition to the mesenchymal phenotype improves the motility of cancer cells and allows them to move easily to other tissues or organs. Abnormal EGFR activation is a well-known signal that stimulates EMT^15–17^ and can be initiated by the release of large amounts of EGFR ligands from tumor cells or nontumor cells in the tumor microenvironment. Among these ligands, heparin-binding EGF-like growth factor (HBEGF) is a well-documented biomarker for many types of cancer, including lung cancer^18^, mucoepidermoid carcinoma^19^, and pancreatic cancer^20^, and is expressed at high levels in macrophages^21–23^. HBEGF is synthesized as a membrane-bound precursor and is then cleaved into a soluble form by matrix metalloproteinases and ADAM family members to activate EGFR and HER4 through autocrine and paracrine routes^24^. EGFR activation by HBEGF may induce EMT in gastric and ovarian cancer^25^, as well as in prostate cancer^26^. HBEGF induces changes in the expression of EMT markers, such as Slug and E-cadherin, in the mouse inner medullary collecting duct^27^. Furthermore, HBEGF contributes to the invasion and metastasis of kidney and bladder cancer cells by increasing cell motility^28^, regulating breast cancer cell migration^29^, and promoting brain metastasis of breast cancer^30^.

In this study, we hypothesized that the inflammatory environment induced by macrophages in response to PM can promote cancer cell metastasis. To test this hypothesis, the effect of PM-induced cytokine production on the motility and metastasis of lung cancer cells was examined using both in vitro and in vivo models, and the underlying mechanisms were investigated.

## Materials and methods

### Cell lines

The mouse melanoma cell line B16F10 was purchased from the Korean Cell Line Bank (KCLB; Seoul, South Korea). The human lung cancer cell line A549 and human monocytic leukemia cell line THP1 were obtained from the American Type Culture Collection (Manassas, VA, USA). The mouse lung carcinoma cell line LLC/luc was provided by Dr Jung-Ki Min (Korea Research Institute of Bioscience and Biotechnology, KRIBB). B16F10 cells were cultured in DMEM (Welgene, Gyeongsan, South Korea), and A549 cells were cultured in RPMI 1640 medium (Welgene, Gyeongsan, South Korea). THP1 cells were cultured in RPMI 1640 medium supplemented with 2-mercaptoethanol at a final concentration of 0.05 mM, and LLC/luc cells were cultured in DMEM supplemented with 2 μg/mL blasticidin (cat. no. ant-bl, InvivoGen, Toulouse, France). All cells were cultured in the presence of 10% fetal bovine serum (RMBIO, Missoula, MT, USA) and 1% antibiotic-antimycotic (cat. no. 15240-062, Gibco, Grand Island, NY) and were maintained at 37 °C in a humidified atmosphere containing 5% CO_2_.

### Antibodies and reagents

Goat anti-rabbit (111-035-045) and goat anti-mouse (115-035-062) antibodies were purchased from Jackson ImmunoResearch Laboratories, Inc. (West Grove, PA, USA). Anti-β-actin (sc-47778) and anti-HBEGF (sc-74441) antibodies were purchased from Santa Cruz Biotechnology (Santa Cruz, CA, USA). The anti-EGFR (ab52894) antibody was purchased from Abcam (Cambridge, United Kingdom), and the anti-Zeb2 (NBP1-82991) antibody was purchased from Novus Biologicals (Minneapolis, MN, USA). Anti-phospho-EGFR (Tyr1068) (#2234), (Tyr1173) (#4407), anti-E-cadherin (#14472), anti-Zeb1 (#3396), anti-Slug (#9585), anti-c-Jun (#9165), anti-phospho-c-Jun (#3270), anti-P65 (#8242), and anti-phospho-P65 (#3033) antibodies were purchased from Cell Signaling Technology (Danvers, MA, USA). Recombinant human HBEGF (cat. no. 100-47) was purchased from Peprotech (Rocky Hill, NJ, USA). The NF-κB inhibitor BAY 11-7082 (cat. no. B5556) was purchased from Sigma-Aldrich (St. Louis, MO, USA), and the AP-1 inhibitor SR 11302 (cat. no. 2476) was purchased from Tocris Bioscience (Ellisville, MO, USA).

### Particulate matter

All PM used in this work was obtained by purchasing the European Reference Materials (ERM-CZ100) product from Sigma-Aldrich (St. Louis, MO, USA) and separating PM10 (fine particles, ≤10 μm) and PM2.5 (≤2.5 μm) by sedimentation. ERM-CZ100 (500 mg) particles were dispersed in ethyl alcohol (100 mL) and sonicated (DH.WUC. A03H, Daihan Scientific, Daegu, Korea) for 15 min. The sonicated PM10 solution was sedimented for 30 min at room temperature. After 30 min, 50 mL of the supernatant was collected and centrifuged at 3220 × *g* for 5 min using a Centrifuge 5810 (Eppendorf, Hamburg, Germany) to separate PM2.5 from the solvent. After removing the supernatant, PM2.5 was collected in a glass vial and dried in a vacuum oven at 80 °C to remove residual ethyl alcohol. Arizona Dust (AD) purchased from Powder Technology Inc. (cat. no. ISO 12103-1, Arden Hills, MN, USA) and Korean road particulate matter (KRPM) were collected from a Dust Suction Road Sweeper Truck.

### Macrophage differentiation and conditioned medium (CM) collection

In total, 4 × 10^6^ THP1 cells were seeded in 6-well plates and incubated with complete medium containing 100 ng/mL phorbol 12-myristate 13-acetate (PMA; Sigma, St. Louis, MO, USA). After 48 h, adherent THP1 cells were washed with phosphate-buffered saline (PBS) and cultured in complete medium. After 24 h, the differentiated THP1 cells were switched to fresh medium and treated with PM at the indicated concentration or for the indicated time, and the culture medium was then collected and centrifuged at 2500 × *g* for 5 min; the same volume of fresh medium was then added, and the solution was used as the CM.

### Generation of human cord blood-derived macrophages

Human cord blood samples were obtained from Chungnam National University Hospital (Daejeon, Republic of Korea; this hospital has received accreditations for cord blood collection and distribution) with the approval of the institutional review board (IRB), and signed informed consent forms were obtained from the donors. To generate human cord blood-derived macrophages, mononuclear cells were isolated from human cord blood using Ficoll-Paque (GE Healthcare Life Science, Rydalmere, Australia) density gradient centrifugation. Then, 2 × 10^7^ monocytes were seeded in 12-well plates and incubated with complete medium containing 50 ng/mL M-CSF (Peprotech, Rocky Hill, NJ, USA) for 7 days.

### siRNA transfection

A TransIT-X2 Dynamic Delivery System (Takara Mirus Bio, Madison, WI) was used for siRNA transfection. siRNA at a final concentration of 100 nM was mixed with Opti-MEM (cat. no. 11058021, Gibco, Grand Island, NY), and TransIT-X2 reagent was then added and incubated for 20 min. The mixture was then added to differentiated THP1 cells. All siRNAs (siP65, 5970-1; sic-Jun, 3725-1; siAhR, 196-1; control siRNA, SN1001) were purchased from Bioneer (Daejeon, South Korea).

### Immunoprecipitation

For immunoprecipitation, the CM of THP1 cells was prepared after PM treatment. Magnetic beads (SureBeads; Bio-Rad, Hercules, CA, USA) were washed three times with PBS containing 0.1% Tween 20 (PBST) and incubated with 10 μg of an anti-HBEGF antibody. After incubation with rotation for 30 min, the magnetic beads were collected, the supernatant was discarded, and the beads were washed three times with PBST. CM was then added to the beads prior to incubation with rotation for 1 h at room temperature (15–25 °C); after rotation overnight at 4 °C, the beads were collected using a magnet, and the supernatant was used as the immunoprecipitate.

### Western blot analysis

Cells were washed with PBS and transferred into tubes. After centrifugation (2500 × g, 5 min), the supernatants were discarded, and the cell pellets were lysed with RIPA buffer (cat. no. CBR002; LPS solution, Daejeon, Republic of Korea) containing protease inhibitor cocktail (cat. no. P3100-005; GenDEPOT, Katy, TX, USA) and phosphatase inhibitor cocktail (cat. no. P3200-005; GenDEPOT, Katy, TX, USA) and incubated on ice. After 20 min, the cell lysates were centrifuged at 16,000 × *g* for 15 min, and the supernatants were then transferred to new tubes. The protein concentrations were then quantified using a BCA protein assay (cat. no. 23225; Thermo Scientific, Waltham, MA, USA). Individual samples were then denatured in 4× sample buffer (cat. no. NP0007; Thermo Scientific) by boiling at 95 °C for 5 min and were then loaded into a 10–12% SDS‒PAGE gel covered with running buffer (cat. no. #1610772; Bio-Rad, Hercules, CA, USA). The separated proteins were transferred to polyvinylidene difluoride membranes (cat. no. IPVH00010; Millipore, Burlington, MA, USA) in transfer buffer (cat. no. #1610771; Bio-Rad, Hercules, CA, USA), blocked with 5% skim milk/PBST for 1 h, and further incubated with the indicated primary antibodies overnight at 4 °C. The membranes were washed three times with PBST and incubated with the secondary antibodies diluted in PBST for 1 h at room temperature (15‒25 °C). The membranes were washed three more times and incubated with Clarity Western ECL substrate (cat. no. #1705061; Bio-Rad, Hercules, CA, USA); the band densities were then determined using a GelDoc XR+ System with Image Lab Software (Bio-Rad, Hercules, CA, USA).

### Immunofluorescence

A549 cells in complete medium were grown on 12-mm cover glasses in 24-well cell culture plates. The cells were then treated with the prepared CM. After 24 h, the cells were washed twice with PBS, fixed with 4% paraformaldehyde for 10 min, and permeabilized with 0.1% Triton X-100 in PBS for 15 min. After blocking with 1% BSA for 1 h, the cells were stained with Alexa Fluor 594 phalloidin for 40 min at room temperature (15‒25 °C). The cells were then washed twice with PBS and mounted with Vectashield mounting medium containing DAPI (cat. no. H-1500; Vector Laboratories, Burlingame, CA, USA); fluorescence images were acquired using a 3D Cell Explorer Fluo microscope (Nanolive, Ecublens, Switzerland).

### Wound healing assay

In total, 7 × 10^5^ A549 cells were cultured in 6-well plates for 12 h. Wounds were made in the confluent cell layers by scratching the cell layers in the vertical and horizontal directions. The cells were then washed twice with PBS and treated with CM. Images were acquired at the indicated time points using a phase contrast microscope (Olympus, Tokyo, Japan). In each image, the width of the scratch was measured at four points along the length of the scratch, and the cell-covered area was quantified using ImageJ (1.53e).

### Cell migration and invasion assays

The cell migration and invasion potential was assessed using a Transwell inserts (cat. no. 353097, Corning Incorporated, Corning, NY, USA). In total, 1 × 10^5^ A549 cells were resuspended in 200 μL of serum-free medium and seeded in the upper compartment of the chamber. Thereafter, 50% of the CM was added to the lower compartment. After incubation for 12 h, the cells that migrated to the lower surface of the insert membrane were fixed with 4% paraformaldehyde for 10 min, washed twice with PBS, and stained with 0.1% crystal violet (Sigma, St. Louis, MO, USA) for 20 min. After two washes, the cells on the upper surface of the membrane in the upper chamber were removed by wiping using cotton swabs. Finally, the cells were imaged using phase contrast microscopy (Olympus CKX53, Tokyo, Japan). For the invasion assay, the upper surface of the Transwell insert membrane was coated with 0.5 mg/mL Matrigel (cat. no. 354234, Corning Incorporated, Corning, NY, USA), and the subsequent procedures were carried out in a manner similar to that used in the migration assay. The migrated and invaded cells were counted in six randomly selected fields.

### Real-time PCR

Total RNA was extracted from cells using the NucleoZOL reagent (cat. no. 740404.200, Macherey-Nagel, Bethlehem, PA, USA) according to the manufacturer’s instructions. For RT‒PCR, cDNA was synthesized using ReverTra Ace qPCR Master Mix with gDNA Remover (cat. no. FSQ-301, TOYOBO, Osaka, Japan). qPCR was performed using specific primers (Supplementary Table 1) in a ROTOR-Gene Q thermocycler (QIAGEN, Hilden, Germany) with THUNDERBIRD SYBR qPCR Mix (cat. no. QPS-201, TOYOBO, Osaka, Japan) and gene-specific primers.

### Cell viability assay

For the cell viability assay, A549 cells were seeded in a 96-well plate at a density of 2 × 10^3^ cells/well. Each group consisted of six replicates. The cells were incubated for 24 h with the indicated concentration of CM before 10 μL of CCK8 reagent (cat. no. CK04-11, Dojindo, Kumamoto, Japan) was added to each well and then incubated for 2 h; the absorbance at a wavelength of 450 nm was then measured using a SpectraMax iD3 multimode microplate reader (Molecular Devices, San Jose, CA, USA).

### Animal experiments

All animal experiments were approved (approval number: KRIBB-AEC-21134) by the Institutional Animal Care and Use Committee of KRIBB (Ochang, Korea). Animal preparation and study design were performed according to the guidelines of the Institutional Animal Care and Use Committee. Seven-week-old male C57BL/6 mice were purchased from DooYeol Biotech (Seoul, Korea) and acclimatized to the laboratory conditions for 1 week. To establish the B16F10 melanoma metastasis model, 3 × 10^5^ cells resuspended in 200 μL of PBS were injected into the tail vein, and 200 μg of PM was delivered into the lung via intratracheal (i.t.) injection daily for 3 days beginning on the day after cell injection. After 10 days, the mice were sacrificed by CO_2_ asphyxiation. For bioluminescence imaging of LLC-luc cells, 2 × 10^6^ cells were injected intravenously, and 200 μg of PM alone or with 5 μg/kg CRM197 (cat. no. 01-515, Bio Academia, Osaka, Japan) was delivered into the lungs of the mice via i.t. injection. After 2 weeks, the mice were injected intraperitoneally (i.p.) with 200 mg/kg body weight D-luciferin (cat. no. 122799, PerkinElmer, Billerica, MA, USA) solution in PBS 10 min prior to imaging. A 3% mixture of isoflurane (Hana Pharm, Kyonggi-Do, Korea) in oxygen was used for introductory anesthesia, and a 1.5% mixture was used for maintenance anesthesia. Bioluminescence measurements were performed using an IVIS Lumina II instrument (Caliper, Australia). Data were acquired and analyzed using Living Image software (version 4.3.1; PerkinElmer).

### RNA sequencing (RNA-seq)

RNA samples were analyzed using an Agilent 2100 Bioanalyzer system (Agilent Technologies). Only high-quality RNA samples (RNA integrity number ≥7.5) were used in the subsequent preparation of mRNA samples for sequencing. An Illumina TruSeq RNA Sample Preparation Kit v2 (Illumina, San Diego, CA, USA) was used with approximately 0.5–4 µg of total RNA to generate the libraries according to the manufacturer’s instructions. Sequencing was performed on the HiSeq2500 platform (Illumina, San Diego, USA) using the standard Illumina RNA-seq protocol by paired-end sequencing with a read length of 100 base pairs.

### Identification of differentially expressed genes (DEGs)

The quality of the sequencing data was evaluated using NGSQCToolkit v2.3.3; adapters were removed using Cutadapt v.1.18 with the default settings, and low-quality sequences were trimmed using Sickle v1.33 with a Phred quality threshold score of 20. A trimmed read was excluded if it contained any ambiguous character (such as N) or was less than 50 bp in length. After preprocessing, the clean reads were mapped to the reference genome (GRCh38) using HISAT2 v2.0.5 with the default parameter settings, and Cufflinks v2.2.1 was used with the reference annotation file to estimate the expression levels of all genes and transcripts as fragments per kilobase of transcript per million mapped reads (FPKM) values. For identification of DEGs, differences in FPKM values calculated by Cuffdiff were considered significant when the *p* value was less than or equal to 0.05 and the absolute fold change value was equal to or greater than 2.

### Functional enrichment analysis

KEGG pathway enrichment analysis was performed with the DEGs with the WebGestaltR v0.4.4 package in R. Pathways with a false discovery rate (FDR) of <0.05 were defined as significantly enriched. We performed gene set enrichment analysis (GSEA) using the gene sets in MSigDB v7.4 (Molecular Signatures Database); the enrichment scores and *p* values were calculated using the default parameters. Genes with FDR *q* value ≤0.05 and adjusted *p* value ≤0.05 were considered to be significantly overrepresented in the ranked gene list.

### Enzyme-linked immunosorbent assay (ELISA)

THP1 cells were treated with each concentration of PM for the indicated times. Supernatants were collected after centrifugation, and the HBEGF concentration was measured using a human HBEGF ELISA kit (cat. no. DY259B; BioLegend, San Diego, CA, USA). After treatment with the TMB substrate reagent (cat. no. 34024; Thermo, Waltham, MA, USA), the absorbance at 450 nm was measured with a SpectraMax iD3 multimode microplate reader (Molecular Devices, San Jose, CA, USA).

### Phospho-RTK array

RTK phosphorylation was quantified using a Human Phospho-RTK Array Kit (cat. no. ARY001B; R&D Systems, Minneapolis, MN, USA). Briefly, A549 cells were cultured in CM from PM-treated THP1 cells (CM-PM) for 24 h and were then rinsed with PBS. A549 cells were lysed in lysis buffer for 30 min on ice. In total, 300 mg of protein was incubated overnight with the phospho-kinase array membrane at 4 °C. The membrane was washed three times with wash buffer and incubated with an HRP-conjugated anti-phosphotyrosine detection antibody for 2 h. Each membrane was washed three times and incubated with Chemi Reagent Mix for 1 min, and quantitation was performed using the GelDoc XR+ System with Image Lab (Bio-Rad, Hercules, CA, USA). For qualitative assessment of the signals, pixel densities were analyzed using ImageJ.

### Statistical analysis

Multidimensional scaling (MDS) analysis was performed using all the protein-coding genes to cluster the samples according to the overall similarity of their gene expression patterns to determine whether the gene expression patterns of the phenotypic classes could be clearly distinguished. For MDS analysis, the pairwise distances between the samples were determined using the function “dist” (maximum distance measure) and were plotted using R. The log_2-_transformed values were used for this analysis, and rows with zero expression in all samples were eliminated. Hierarchical clustering was performed, and a heatmap was generated using “heatmap.2” (ward.D2 clustering function) in the gplots package (v3.1.1) to determine the phenotypic grouping and the specific gene expression patterns. All experiments were performed in triplicate. Data are presented as the mean ± SD values. Data were processed in Microsoft Office Excel 2016 and GraphPad Prism software version 8.1.1 (Prism, La Jolla, CA, USA). Statistical analyses were performed using *t* test*s*. Statistical significance was set at *P* ≤ 0.05.

## Results

### PM exposure changes the characteristics of macrophages

**Fig. 1:**
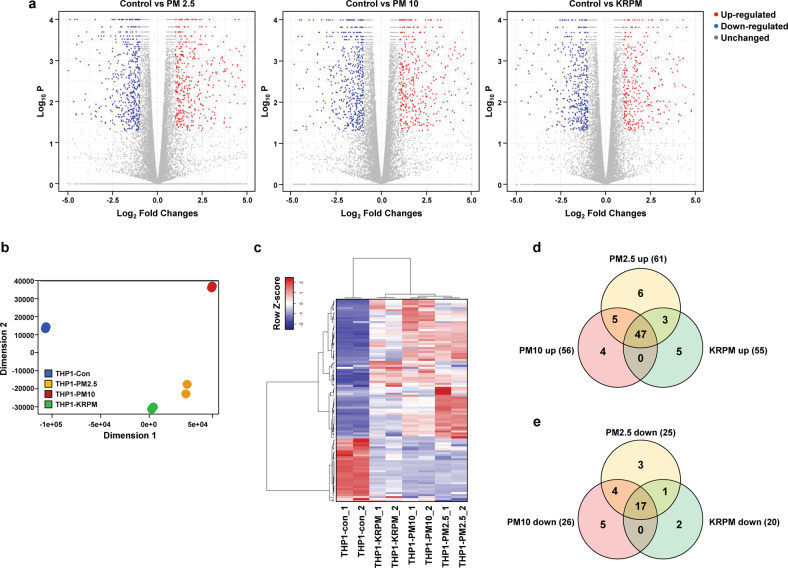
PM exposure changes the characteristics of macrophages. **a** Volcano plots of differentially expressed genes in THP1 cells after treatment with the indicated PM types for 24 h. **b** Multidimensional scaling (MDS) plot of RNA-seq expression profiles in two dimensions. **c** Heatmap of 452 differentially expressed human cytokine and growth factor genes between THP1-Control cells and THP1-PM2.5, THP1-PM10, and THP1-KRPM cells identified by RNA-seq. **d** Venn diagram of upregulated cytokines and growth factors. **e** Venn diagram of downregulated cytokines and growth factors.

PM inhaled into the lungs is internalized by macrophages, which show various responses. To investigate the characteristics of macrophages changed by PM exposure, RNA-seq was performed on THP1 cells after treatment with PM2.5 (separated from PM10; Supplementary Fig. 1a–d), PM10 and KRPM (PM from Korean roads; Supplementary Fig. 1e, f). This analysis showed a high correlation between the two biological replicates (Supplementary Fig. 2a). PM2.5 treatment increased and decreased the expression levels of 1386 and 1155 genes, respectively, by more than 2-fold compared to the corresponding levels in the control group. In addition, the expression levels of 1192 and 1175 genes were increased and decreased, respectively, by PM10 treatment, and those of 900 and 886 genes were increased and decreased by KRPM treatment. The expression levels of a total of 3130 genes were significantly changed, with 756 genes showing increased expression and 654 genes showing decreased expression after exposure to each of the three types of PM (Fig. 1a and Supplementary Fig. 2b–d). Multidimensional scaling (MDS) analysis showed that the clusters for the three types of PM were decoupled from the control cluster (Fig. 1b), suggesting that PM greatly modulates the gene expression dynamics in macrophages and causes global changes. In addition, PM altered the secretion of various proteins by macrophages. Upon comparing the changes in the treated groups compared with the control group, among the genes encoding the 452 differentially expressed human cytokines and growth factors (Fig. 1c), 47 and 17 genes were increased and decreased, respectively, after exposure to each of the three types of PM (Fig. 1d, e). Cytokines and growth factors secreted from macrophages can stimulate other cells through various pathways, particularly affecting the motility of cancer cells. These results suggest that the expression of cytokines and growth factors was increased in macrophages via PM stimulation, which in turn can induce changes in cancer cell motility.

### CM from PM-treated macrophages increases cancer cell motility and activates EGFR in cancer cells

**Fig. 2:**
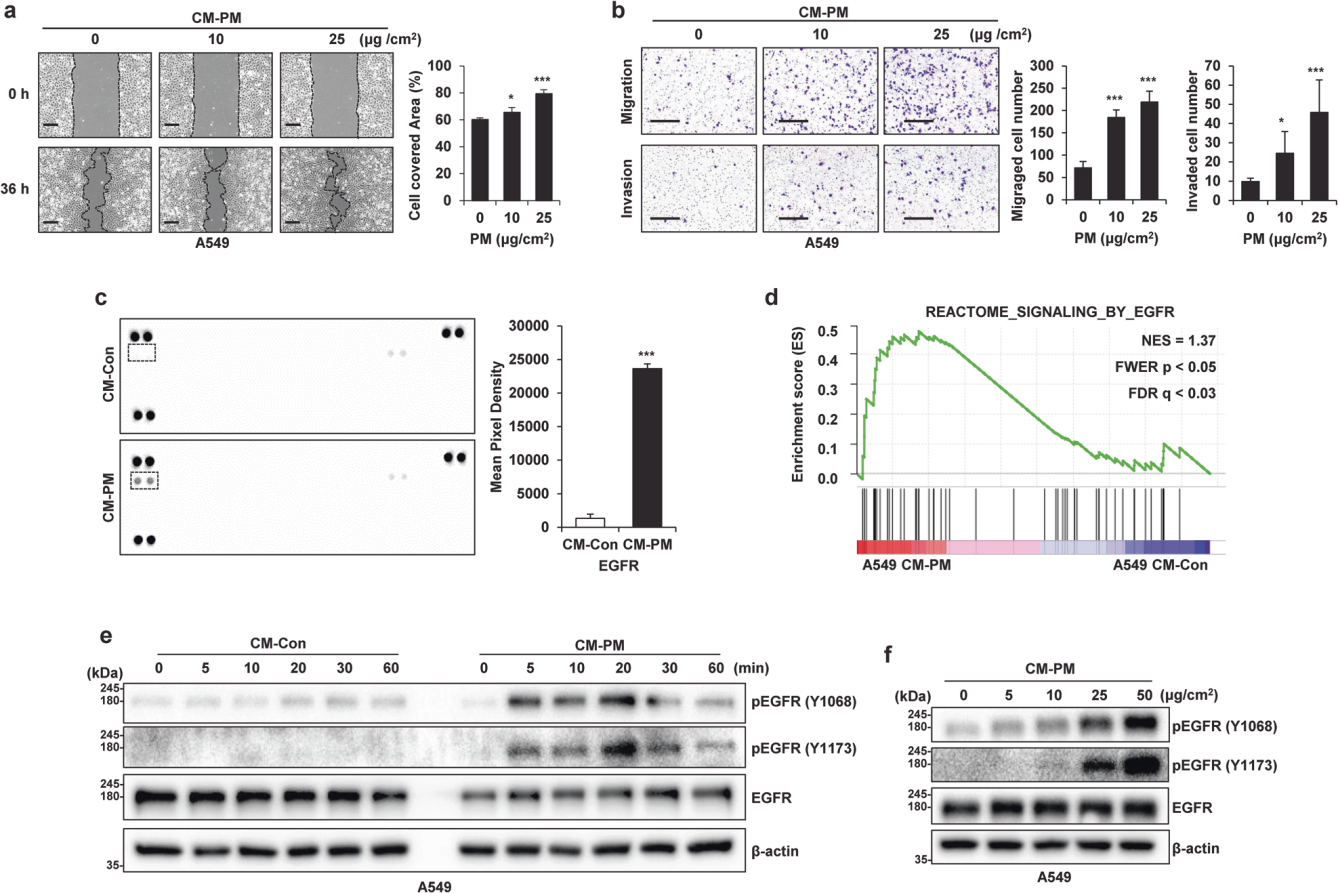
Conditioned medium (CM) from particulate matter (PM)-treated macrophages increases cancer cell motility and activates EGFR expression in cancer cells. **a** Wound healing assay of A549 cells incubated with CM from PM-treated THP1 cells; images were acquired at 0 and 36 h. Scale bar, 250 μm. **b** Transwell migration (top) and invasion (bottom) assays of A549 cells incubated with CM from PM-treated THP1 cells. Scale bar, 200 μm. **c** Phospho-RTK array assay of A549 CM-Control (Con) and A549 CM-PM cells. The dots inside the rectangle represent EGFR (left). Relative pixel intensities indicating phospho-EGFR levels in the phospho-RTK array (right). **d** Gene set enrichment analysis plot of EGFR-induced genes in A549 CM-PM cells. NES normalized enrichment score, FWER familywise error rate, FDR false discovery rate. **e** Immunoblot analysis of EGFR phosphorylation in A549 cells after incubation with CM from PM-treated THP1 cells for the indicated time. β-Actin was used as the loading control. **f** Immunoblot analysis of EGFR phosphorylation in A549 cells after incubation with the indicated concentrations of CM from PM-treated THP1 cells at for 20 min. *P ≤ 0.05, ***P ≤ 0.001.

Since the PM-induced increases in the expression of cytokines and growth factors in macrophages are related to cancer malignancy, we investigated whether treating cancer cells with conditioned medium from macrophages treated with PM (CM-PM) increases their motility. Our results confirmed that the motility of cancer cells was indeed increased by CM-PM treatment (Fig. 2a, b and Supplementary Fig. 3b, c). Furthermore, treatment of cancer cells with CM-PM did not increase their growth (Supplementary Fig. 3a). Receptor tyrosine kinases (RTKs) are closely related to many growth factors and cytokines and play an important role in cell motility^31^. To analyze the signaling molecules in CM-PM, tyrosine phosphorylation by 49 RTKs in A549 cells was compared using an antibody-based array. The results confirmed that tyrosine phosphorylation of EGFR was significantly increased in A549 cells treated with CM-PM (A549 CM-PM) (Fig. 2c), and that EGFR-related genes were significantly upregulated in response to CM-PM, as shown in the gene set enrichment analysis (GSEA) plots (Fig. 2d). Furthermore, EGFR expression was strongly stimulated within 20 min after CM-PM treatment (Fig. 2e) in a PM concentration-dependent manner (Fig. 2f).

### HBEGF secreted by PM-treated macrophages activates EGFR in cancer cells

**Fig. 3:**
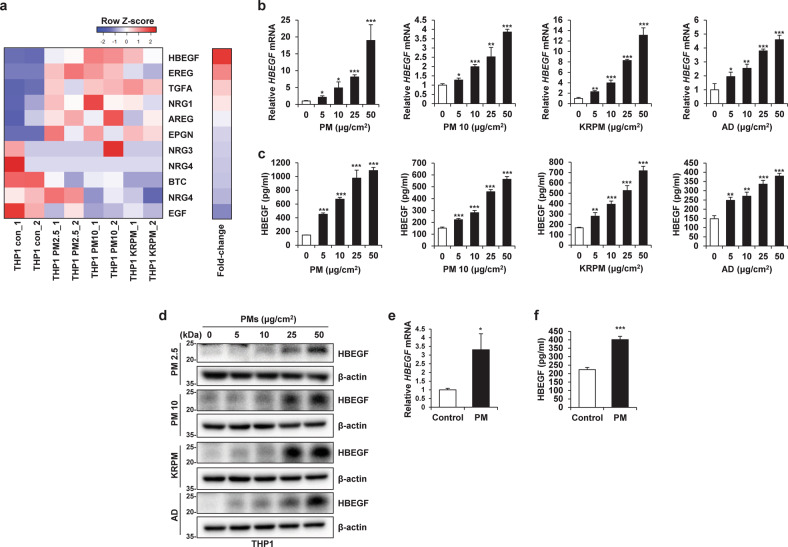
Particulate matter (PM)-treated macrophages exhibit increased HBEGF expression. **a** Heatmap showing the differential expression of the indicated EGFR ligands between THP1-Control (Con) cells and THP1-PM2.5, THP1-PM10, and THP1-KRPM cells identified by RNA-seq. **b** qRT‒PCR analysis of THP1 cells treated with the indicated concentrations of different types of PM for 24 h. **c** ELISA of THP1 cells treated with the indicated concentrations of different types of PM for 24 h. AD Arizona dust. **d** Immunoblot analysis of THP1 cells treated with the indicated concentrations of different types of PM for 24 h. **e** qRT‒PCR analysis of human cord blood-derived macrophages treated with 5 μg/cm^2^ PM for 24 h. **f** ELISA of human cord blood-derived macrophages treated with 5 μg/cm^2^ PM for 24 h. *P ≤ 0.05, **P ≤ 0.01, ***P ≤ 0.001.

**Fig. 4:**
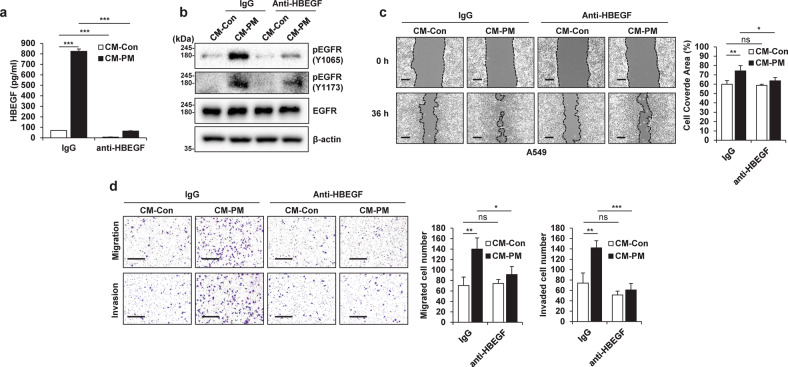
HBEGF secreted by particulate matter (PM)-treated macrophages increases cancer cell motility and activates EGFR in cancer cells. **a** ELISA of conditioned medium from particulate matter-treated cells (CM-PM) immunoprecipitated with control IgG or an anti-HBEGF antibody. **b** Immunoblot analysis of EGFR phosphorylation in A549 cells after treatment with CM-PM immunoprecipitated with control IgG or an anti-HBEGF antibody. **c** Wound healing assay of A549 cells treated with CM-PM immunoprecipitated using control IgG or an anti-HBEGF antibody. Images were acquired at 0 and 36 h. Scale bar, 250 μm. **d** Transwell migration (top) and invasion (bottom) assays of A549 cells treated with CM-PM immunoprecipitated using control IgG or an anti-HBEGF antibody. Scale bar, 200 μm. *P ≤ 0.05, **P ≤ 0.01, ***P ≤ 0.001; ns not significant.

Upon analysis of the changes in the expression of EGFR ligands that can stimulate EGFR expression in A549 cells, the expression of several EGFR ligands was found to be significantly increased (Supplementary Fig. 4a) in PM-treated THP1 (THP1-PM) cells compared with control THP1 (THP1-Con) cells. In particular, HBEGF expression showed the greatest increase in THP1-PM cells (Fig. 3a and Supplementary Fig. 4b). We further confirmed that the RNA and protein expression of HBEGF was increased by the various types of PM and that HBEGF secretion increased in a dose- and time-dependent manner (Fig. 3b–d and Supplementary Fig. 4c–e). Moreover, human cord blood-derived macrophages expressed and secreted HBEGF in response to PM (Fig. 3e, f). Notably, the increase in HBEGF caused by PM was independent of the particle size and was not a unique effect caused by a specific type of particulate matter; rather, it was observed as a common effect of exposure to several different types of PM. HBEGF is known to activate EGFR and HER4^32^; however, we could not confirm the activation of HER4 in A549 CM-PM cells (Fig. 2c and Supplementary Fig. 4f, g). To determine whether the increased level of HBEGF in PM-stimulated THP1 cells mediates the increase in the motility of cancer cells, we treated A549 CM-Con cells with recombinant human HBEGF (rhHBEGF). We found that EGFR in A549 cells was effectively activated by adding only rhHBEGF to A549 CM-Con cells (Supplementary Fig. 5a). Furthermore, the wound healing, Transwell migration, and invasion abilities of A549 cells were enhanced with increasing rhHBEGF concentration (Supplementary Fig. 5b, c). When only HBEGF was removed from CM-PM via immunoprecipitation (Fig. 4a), EGFR activation in A549 cells was decreased (Fig. 4b). This removal further suppressed the increase in motility (Fig. 4c, d). Overall, these results indicated that PM-stimulated macrophages showed increased expression of HBEGF, which stimulated EGFR on cancer cells and increased their motility.

### CM from PM-treated macrophages induces EMT in cancer cells

**Fig. 5:**
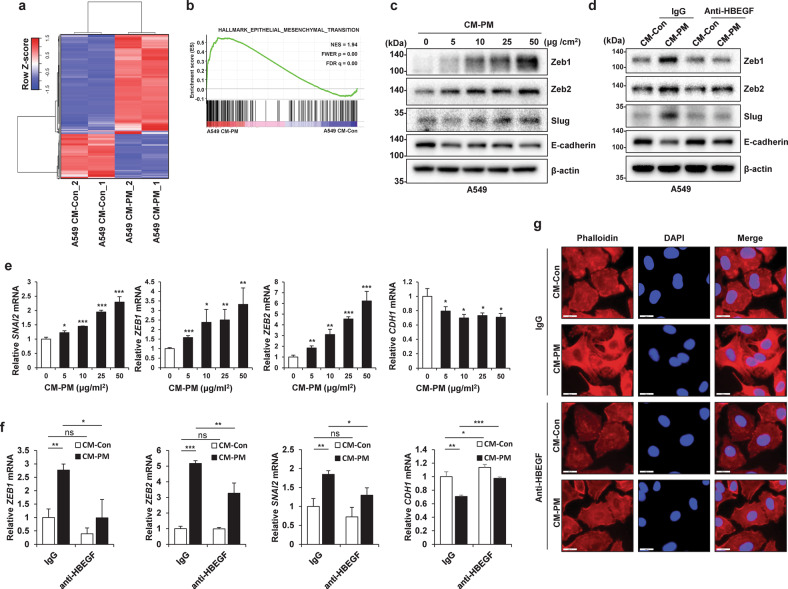
Conditioned medium (CM) from particulate matter (PM)-treated macrophages induces epithelial-to-mesenchymal transition (EMT) in cancer cells. **a** Heatmap of differential gene expression between A549 CM-Control (Con) and A549 CM-PM cells identified by RNA-seq. **b** Gene set enrichment analysis plot for the “Epithelial-to-mesenchymal transition” gene set from the HALLMARK gene collection. NES normalized enrichment score; FWER familywise error rate; FDR false discovery rate. **c** Immunoblot analysis of A549 cells incubated for 24 h with the indicated concentration of CM from PM-treated THP1 cells. β-Actin was used as the loading control. **d** Immunoblot analysis of A549 cells treated with CM-PM immunoprecipitated using control IgG or an anti-HBEGF antibody for 24 h. **e** qRT‒PCR analysis of A549 cells incubated for 24 h with the indicated concentration of CM from PM-treated THP1 cells. **f** qRT‒PCR analysis of A549 cells treated with CM-PM immunoprecipitated using control IgG or an anti-HBEGF antibody for 24 h. **g** Immunofluorescence analysis of A549 cells cultured with CM-PM immunoprecipitated using control IgG or an anti-HBEGF antibody for 24 h. Scale bar, 20 μm. *P ≤ 0.05, **P ≤ 0.01, ***P ≤ 0.001; ns not significant.

To obtain a broad perspective on the effect of PM-stimulated macrophages on cancer cells, we performed RNA-seq analysis with two biological replicates of two groups of A549 cells, i.e., A549 CM-Con cells and A549 CM-PM cells. Both replicates showed a high correlation (Supplementary Fig. 2e). Among 19,899 protein-coding genes, 1051 differentially expressed genes (DEGs)—specifically, 721 (68.6%) upregulated and 330 (31.4%) downregulated genes—were identified by differential gene expression analysis between A549 CM-PM and A549 CM-Con cells (Fig. 5a). There are at least three signaling pathways downstream of EGFR, namely, the PI3K/AKT, JAK/STAT, and MAPK/MEK/ERK pathways, all of which are related to EMT^33^ and were found to be activated in CM-PM-treated A549 cells in our study (Supplementary Fig. 6a, b). GSEA showed that in A549 cells, EMT-related genes were significantly upregulated in response to CM-PM treatment (Fig. 5b). Furthermore, CM-PM increased the expression of the mesenchymal markers Slug, Zeb1, and Zeb2 and reduced the expression of the epithelial marker E-cadherin in cancer cells at both the transcriptional and translational levels (Fig. 5c, e). To confirm the association of HBEGF with the increase in CM-PM-induced EMT, rhHBEGF was added to CM-Con and used to treat A549 cells. The results confirmed that in these cells, the mesenchymal markers Slug, Zeb1, and Zeb2 were upregulated, whereas the epithelial marker E-cadherin was downregulated (Supplementary Fig. 7a, b). When HBEGF in was removed from CM by immunoprecipitation, the expression of the mesenchymal markers decreased, and that of E-cadherin increased (Fig. 5d, f). In addition, F-actin staining confirmed that the addition of CM-Con combined with rhHBEGF changed the morphology of A549 cells to a mesenchymal phenotype (Supplementary Fig. 7c). Furthermore, when HBEGF was removed from CM-PM, the morphology of A549 cells was confirmed to maintain an epithelial phenotype (Fig. 5g). This is a typical characteristic of EMT^34^ and indicates that HBEGF is a key factor in cancer cell EMT induced by PM-stimulated macrophages.

### Aryl hydrocarbon receptor (AhR) activation by PM in macrophages increases HBEGF expression

**Fig. 6:**
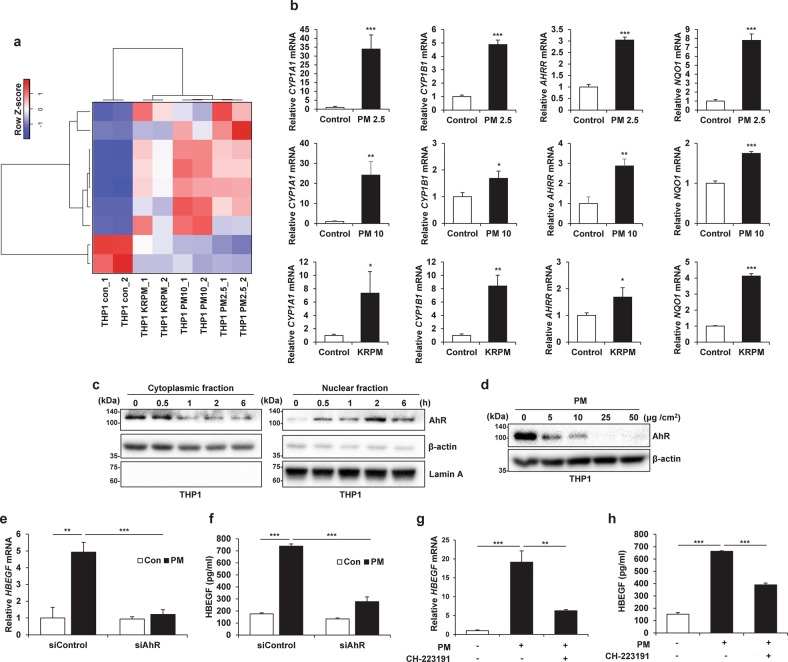
Activation of the aryl hydrocarbon receptor (AhR) by particulate matter (PM) in macrophages increases HBEGF expression. **a** Heatmap of the differential expression of AhR-related genes between THP1-Control (Con) cells and THP1-PM2.5, THP1-PM10, and THP1-KRPM cells identified by RNA-seq. **b** qRT‒PCR analysis of THP1 cells treated with different types of PM (25 μg/cm^2^) for 24 h. **c** Immunoblot analysis of PM2.5 (25 μg/cm^2^)-stimulated AhR translocation. β-Actin and Lamin A were used as the cytoplasmic fraction and nuclear fraction loading controls, respectively. **d** Immunoblot analysis of THP1 cells treated with the indicated concentrations of PM2.5 for 24 h. **e**, **f** qRT-PCR (**e**) and ELISA (**f**) of THP1 cells transfected with 50 nM siAhR and treated with 25 μg/cm^2^ PM2.5 for 24 h. **g**, **h** qRT‒PCR (**g**) and ELISA (**h**) of THP1 cells pretreated with 10 nM CH-223191 for 1 h and treated with 25 μg/cm^2^ PM2.5 for an additional 24 h. *P ≤ 0.05, **P ≤ 0.01, ***P ≤ 0.001; ns not significant.

The RNA-seq data confirmed that AhR signaling in THP1 cells was significantly activated by PM exposure (Fig. 6a). Furthermore, treatment of THP1 cells with PM increased the expression of AhR target genes (Fig. 6b). Moreover, treatment of human cord blood-derived macrophages with PM increased the expression of AhR target genes (Supplementary Fig. 8). AhR is localized in the cytoplasm and is translocated to the nucleus upon ligand binding, dimerizing with the AhR nuclear translocator and binding to xenobiotic-responsive elements in AhR target gene promoters^35^. Nuclear translocation of AhR occurred in a time-dependent manner in our study (Fig. 6c). AhR is reported to be controlled by the ubiquitin‒proteasome pathway^36^. To further confirm AhR activation by PM, we measured the AhR protein level in THP1 cells after PM treatment and found that the AhR protein level decreased in a dose-dependent manner (Fig. 6d). As reported previously, AhR, as well as NF-κB and AP-1, can be activated by PM^37–39^. Accordingly, NF-κB and AP-1 signaling was also activated by PM (Supplementary Fig. 9a–d). However, when we searched the target genes of these transcription factors in the TRRUST version 2 database, we found that among the signaling mediated by these three transcription factors, AhR signaling was the most activated (Supplementary Fig. 9e). As these transcription factors can increase the transcription of HBEGF^40^, we suppressed these transcription factors to determine whether their activation can increase HBEGF expression. When AhR was suppressed, the increase in HBEGF expression was significantly suppressed even with PM treatment (Fig. 6e, f). Compared to suppression of the other two transcription factors, suppression of AhR exerted a markedly weaker effect on reducing the HBEGF level (Supplementary Fig. 9f). Furthermore, treatment of THP1 cells with CH-223191, an AhR antagonist, inhibited the increase in HBEGF expression upon PM treatment (Fig. 6g, h). These results indicated that HBEGF transcription is regulated mainly by AhR in response to PM.

### The increase in the HBEGF levels induced by PM contributes to lung cancer metastasis in mice

**Fig. 7:**
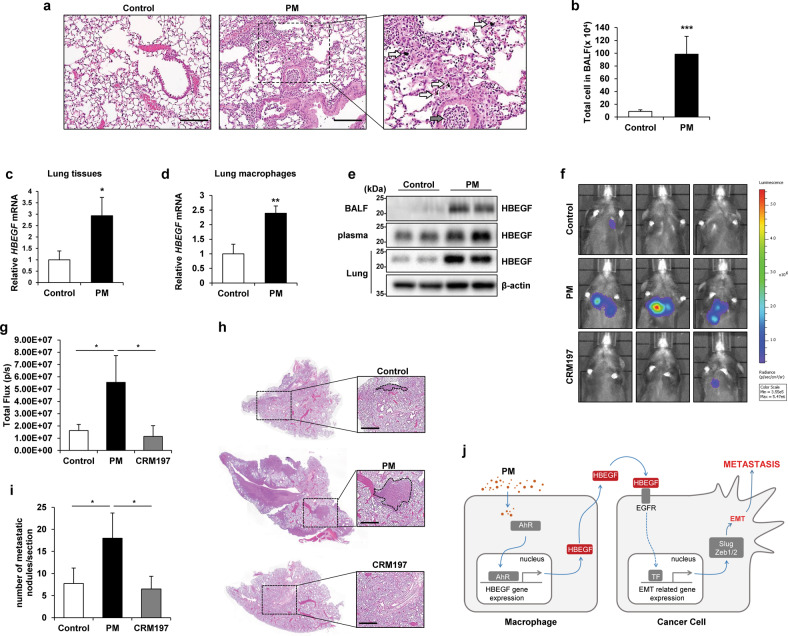
The increase in the HBEGF level induced by particulate matter (PM) contributes to lung cancer metastasis in mice. **a** Hematoxylin and eosin (H&E) staining of lung samples harvested from control and PM-treated mice. Black arrow, immune cells; white arrow, PM. Scale bar, 100 μm. **b** Quantification of total cells in the bronchoalveolar lavage fluid (BALF) of control and PM-treated mice. **c** qRT‒PCR analysis of whole lungs from control and PM-treated mice. **d** qRT‒PCR analysis of macrophages from the lungs of control and PM-treated mice. **e** Immunoblot analysis of BALF (top line), plasma (middle line), and whole-lung lysate (bottom line) from control and PM-treated mice. β-Actin was used as the loading control for the whole-lung lysate. **f** In vivo metastasis assay with intravenously injected luciferase-expressing Lewis lung carcinoma (LLC-luc) cells; 24 h after intravenous injection of cells, mice were injected intratracheally three times over 3 days with PM alone or with CRM197 (an inhibitor of HBEGF). IVIS images were acquired 14 days after intravenous injection of cells. **g** Quantification of bioluminescence (*n* = 5 mice per group). **h** Representative images of H&E staining from the in vivo metastasis assay. Scale bar, 500 μm. **i** Quantification of metastatic lung nodules. **j** Proposed model of metastasis with PM-exposed macrophages. PM induces HBEGF expression in macrophages through activation of NF-κB and AP-1, which promotes EMT in cancer cells and facilitates metastasis. *P ≤ 0.05, **P ≤ 0.01, ***P ≤ 0.001; ns not significant.

Previous studies have shown that PM inhaled into the lungs recruits immune cells^41^. Here, when PM was introduced into the lungs through intratracheal (i.t.) injection, it was deposited in the lung tissue, and the infiltration of inflammatory cells into the bronchioles and alveolar tissues was confirmed (Fig. 7a). In addition, the total number of cells in the bronchoalveolar lavage fluid (BALF) of mice injected with PM was increased (Fig. 7b). To confirm the PM-induced changes in the metastatic ability of cancer cells, we injected B16F10 mouse melanoma cells, an established model of metastasis^42^, into mice via the tail vein, and beginning on the subsequent day, injected PM into the lungs for 3 days (Supplementary Fig. 10a). The results confirmed that lung metastasis of B16F10 cells was increased by PM exposure (Supplementary Fig. 10b–d). To determine whether the lungs of mice exposed to PM harbored an environment with increased HBEGF expression, mice were subjected to i.t. injection of PM into the lungs and were sacrificed after 12 h to determine the environmental changes. First, we confirmed that HBEGF mRNA expression was increased in lung tissues (Fig. 7c). In particular, it was confirmed that HBEGF expression was increased only in macrophages isolated from the lungs of mice treated with PM (Fig. 7d). Furthermore, the HBEGF protein level was increased in BALF, plasma, and lung lysates (Fig. 7e). Next, to examine the effect of PM on the metastatic ability of cancer cells, luciferase-expressing Lewis lung carcinoma (LLC-luc) cells were injected intravenously into C57BL/6 mice. Beginning on the subsequent day, the mice were treated with PM alone or with CRM197, an inhibitor of HBEGF, via i.t. injection into the lungs for 3 days. The results confirmed that lung cancer metastasis was increased by PM and decreased by cotreatment with PM and CRM197 (Fig. 7f–i). Taken together, these data implied that PM induces an increase in HBEGF expression in mouse lungs, which may in turn increase lung cancer metastasis.

## Discussion

In this study, we investigated the effects of PM exposure on cancer metastasis and demonstrated that PM induces HBEGF expression in macrophages and that secreted HBEGF is crucial for mediating cancer cell metastasis through EMT (Fig. 7j). This increase in HBEGF expression was confirmed in immortalized cell lines as well as in human cord blood-derived macrophages, suggesting that PM can induce an increase in HBEGF expression in humans. EGFR ligands, including HBEGF, increase the protein expression of snail, slug, and twist, causing a decrease in E-cadherin expression in cancer cells^43^. Moreover, HBEGF can regulate EMT in human keratinocytes^44^ and contribute to the severity of chronic obstructive pulmonary disease by regulating pulmonary EMT^45^. It has been reported that tissue-resident synovial fibroblasts shape HBEGF^+^ inflammatory macrophages and promote fibroblast invasiveness through the EGFR response^46^. These findings indicate the direct effect of macrophage-secreted HBEGF on cancer EMT, which greatly extends our understanding of PM-induced cancer metastasis.

In our study, HBEGF transcription was increased by AhR in response to PM. AhR is a ligand-activated transcription factor and member of the periodic circadian protein (PER)–nuclear translocator (ARNT)–single-handed protein (SIM) superfamily. AhR is activated by endogenous factors such as oxygen tension or redox potential or by exogenous factors such as polyaromatic hydrocarbons (PAHs) and environmental toxins^35^. The certificate of analysis issued by the European Commission indicates that ERM-CZ100, the particulate matter we used, contains three major PAHs (pyrene, fluoranthene, and anthracene). This composition was confirmed by GC‒MS analysis. In addition, the organic components of KRPM were analyzed by GC‒MS using a DB-5MS column to confirm the presence of meaningful amounts of PAHs. Since an increase in HBEGF gene expression has also been reported in PAH-treated C10 cells^47^, it can be concluded that the activation of AhR by PM regulates HBEGF transcription. Although the PM component that increased AhR activation in macrophages was not identified, PM is composed of several substances that can activate AhR and induce an increase in HBEGF expression.

Furthermore, NF-κB and AP-1, well-known transcription factors mediating HBEGF activation^40^, were also activated by PM. These two transcription factor signaling pathways were found to be activated within minutes by PM in our study (Supplementary Fig. 5c). The endotoxin component of PM may activate NF-κB and AP-1 through the TLR4/MyD88 signaling pathway^9^; hence, they may also be involved in HBEGF expression independent of AhR. Moreover, the level of intracellular free calcium ions has been shown to be increased by activated AhR, thereby activating CaMKII and in turn AP-1, revealing another mechanism through which HBEGF expression could be increased^48^. Furthermore, AhR and p65 may directly interact to activate NF-κB signaling^49^. Collectively, our findings demonstrated that PM-stimulated AhR has a crucial effect on HBEGF expression and that NF-κB and AP-1 are also involved in this process.

Considering that direct treatment with PM (data not shown) or CM (Supplementary Fig. 3a) does not increase cancer cell viability, the increase in lung cancer metastasis can be attributed to improved cell motility. Previous studies have shown that PM can directly regulate the motility of non-small cell lung cancer cells. For instance, cancer cell motility is increased when cancer cells are subjected to direct treatment with PM followed by PM removal^50^ or upon treatment with a low concentration of PM^51^. However, we found that PM did not significantly change the motility of cancer cells; rather, high concentrations of PM inhibited the motility of cancer cells due to physical inhibition. In addition, the effect of direct treatment with PM on the expression of HBEGF in cancer cells remains to be determined. PM may be indirectly involved in cancer cell motility; we found that macrophages act as mediators to transmit signals induced by PM to cancer cells.

PM stimulates bronchial/alveolar epithelial cells to initiate an immune response and induces cytokine expression in primary human bronchial epithelial cells^52^. In particular, an increase in the level of HBEGF as well as amphiregulin, TGFα, and BTC was confirmed when the 16HBE human bronchial epithelial cell line was treated with PM2.5^53^. However, macrophages are innate immune cells that serve as the first line of defense against inhaled PM in the small (lower) airway. Furthermore, as exposure to PM promotes monocyte release from the bone marrow and increases their lung recruitment and differentiation into macrophages^54^, increased HBEGF expression in macrophages is important. Collectively, our findings indicate that an environment of EGFR activation is generated by the total increase in EGFR ligand expression by lung cells in lungs after PM inhalation, which can increase cancer metastasis.

Our study demonstrates the mechanism underlying PM-induced cancer metastasis and the role of macrophages in cancer cell migration through HBEGF-mediated signaling and suggests that HBEGF expression in macrophages plays a key role in PM-induced cancer metastasis and may serve as a prognostic marker and target to prevent lung metastasis of cancer.

## Supplementary information

Supplementary figures and table
