# Supplementary material for: Particulate matter promotes cancer metastasis through increased HBEGF expression in macrophages
Source: Exp Mol Med. 2022 Nov 9;54(11):1901–12. doi: 10.1038/s12276-022-00886-x (PMC9722902; doi:10.1038/s12276-022-00886-x)
Supplement: Supplementary file 1 — Supplementary figures and table [file 12276_2022_886_MOESM1_ESM.pdf]

Supplementary Information

Particulate matter promotes cancer metastasis through increased HBEGF expression in macrophages

Seung-Ho Park[*], Sung-Jin Yoon[*], Song Choi[*], Jaeeun Jung, Jun-Young Park, Young-Ho Park, Jinho Seo, Jungwoon Lee, Moo-Seung Lee[6], Seon-Jin Lee, Mi-Young Son, Young-Lai Cho, Jang-Seong Kim, Hyo Jin Lee, Jinyoung Jeong[#], Dae-Soo Kim[#] and Young-Jun Park[#]

[*]These authors contributed equally to this work.

[#]Corresponding authors.

This file contains Supplementary Figures 1–10 and Supplementary Table 1.

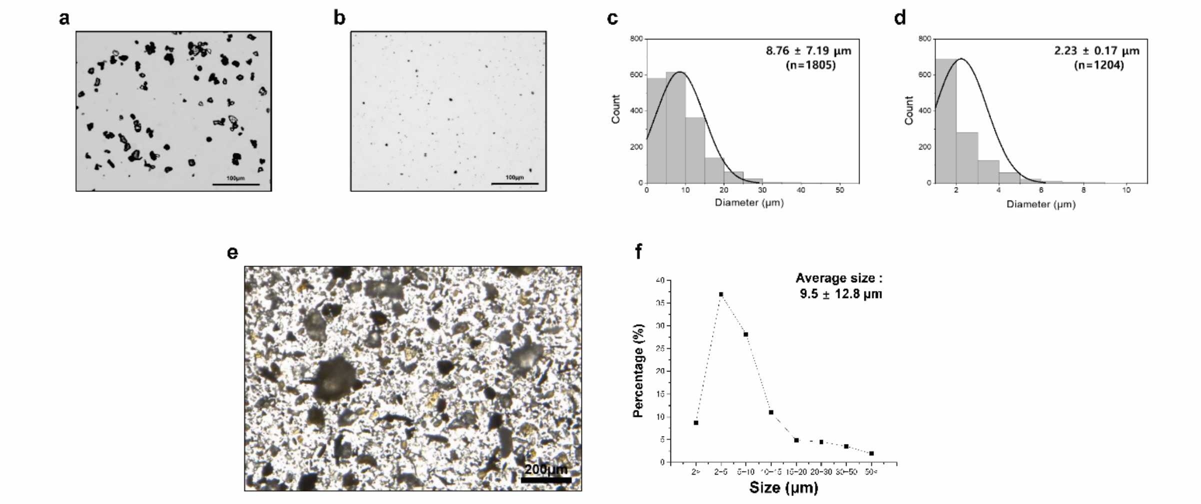

Supplementary Fig. 1 The characteristics of PMs used in this study. **a, b** Optical images of PM10 (a) and PM2.5 (b). Scale bar, 100 µm. **c, d** Size distribution of PM10 (c) and PM2.5(d). **e** Optical image of KRPM. **f** Size distribution of KRPM.

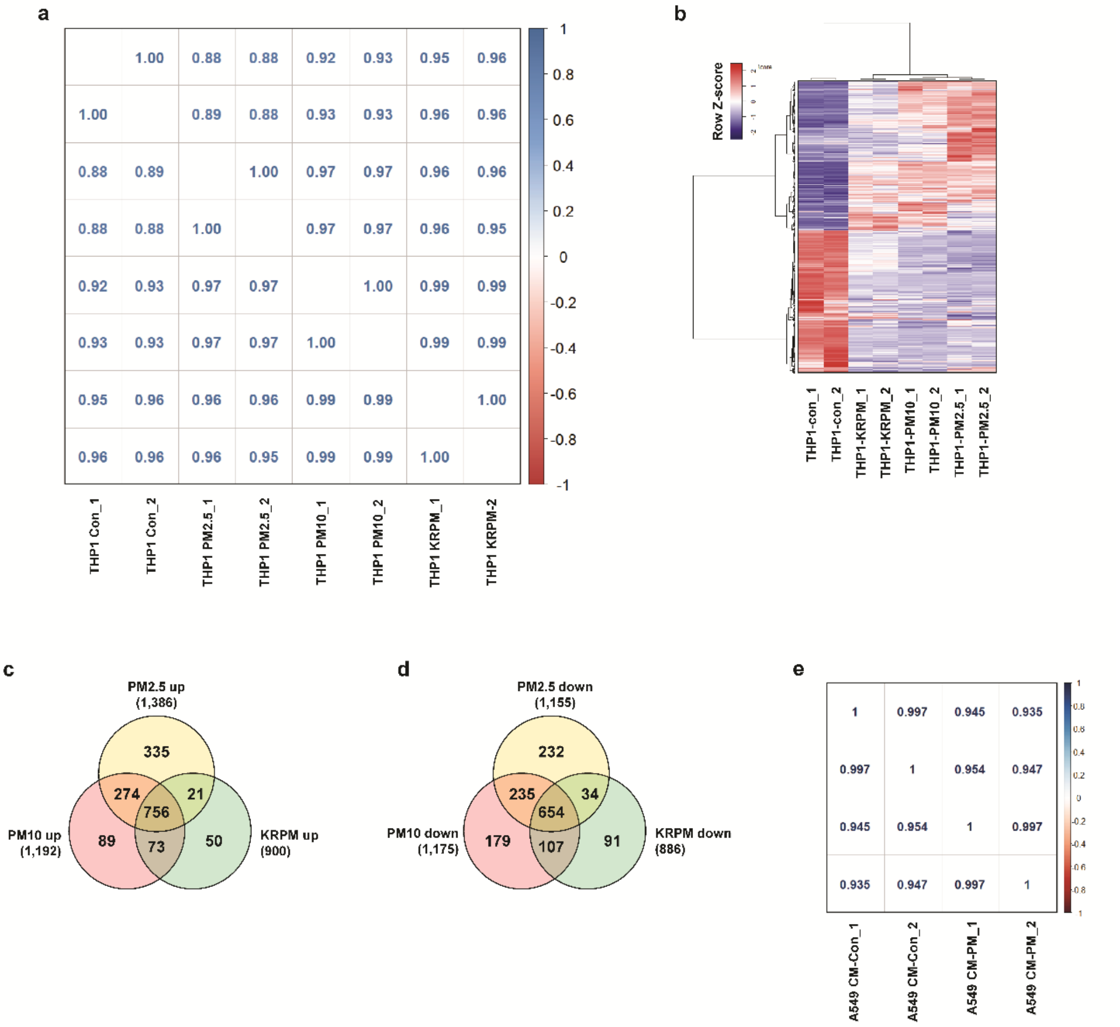

Supplementary Fig. 2 Biological RNA samples showed high correlation. **a** Pearson correlation analysis between THP1-Control (Con) and THP1-PM2.5, THP1-PM10, THP1-KRPM (Particulate matter from Korea roads). **b** Heatmap profiles of differential gene expression between THP1-Con and THP1-PM2.5, THP1-PM10, THP1-KRPM from RNA-seq datasets. **c, d** Venn diagram of upregulated genes from RNA-seq datasets (c), and downregulated genes from RNA-seq datasets (d). **e** Pearson correlation analysis between A549 CM-Con and A549 Conditioned media from particulate matter treated cells (CM-PM).

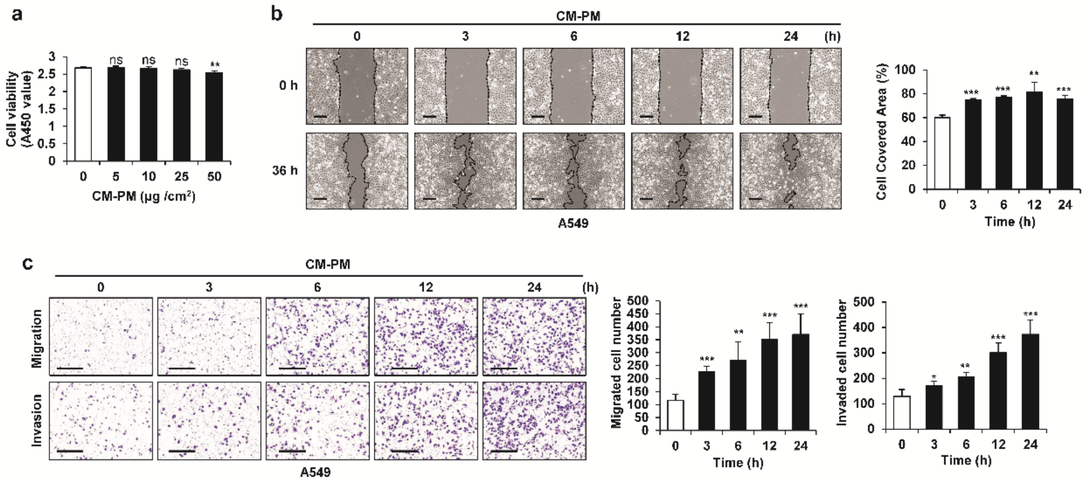

Supplementary Fig. 3 Conditioned media (CM) from THP1 cells treated with PM (CM-PM) increases the motility of cancer cells in a time-dependent manner. **a** CCK8 assay of CM-treated A549 cells, measured 24 h after CM treatment. **b** Wound healing assay of A549 cells treated with the indicated concentrations of PM. Images were captured at time 0 and 36 h. Scale bar, 250 μm. **c** Transwell migration (top) and invasion (bottom) assays of A549 cells treated with CM-PM at the indicated times. Scale bar, 200 μm. *$P \leq 0.05$, **$P \leq 0.01$, ***$P \leq 0.001$; ns, not significant.

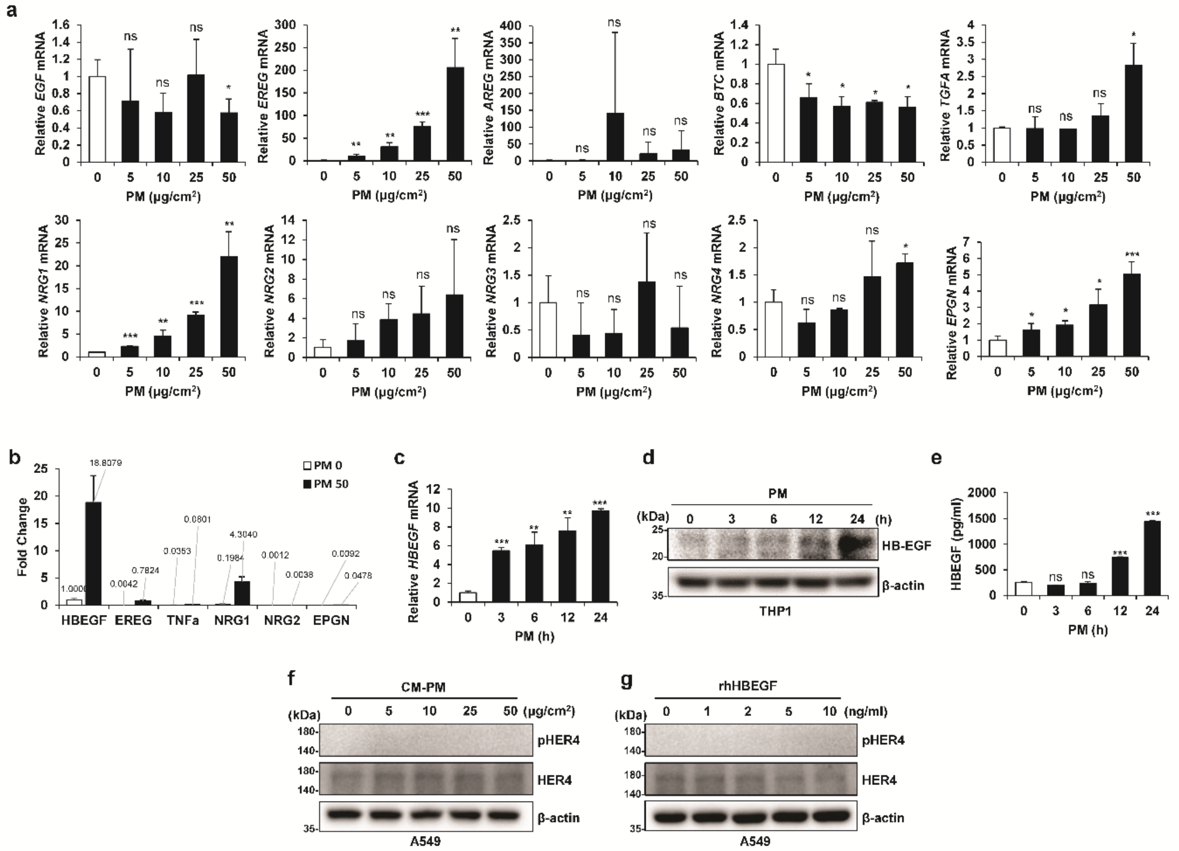

Supplementary Fig. 4 Expression of other EGFR ligands in THP1 cells treated with particulate matter (PM). **a** qRT-PCR analysis of THP1 cells treated with the indicated concentration of PM for 24 h. **b** Comparison of expression levels through qRT-PCR between HBEGF and other EGFR ligands. HBEGF levels in untreated PM were used as the control. Numbers indicate fold change compared to the control. **c** qRT-PCR analysis of THP1 treated with PM (25 μg/cm^2) for the indicated time. **d** Immunoblot analysis of THP1 treated with PM (25 μg/cm^2) for the indicated time. β-Actin was used as the loading control. **e** ELISA of THP1 treated with PM (25 μg/cm^2) for the indicated time. **f** Immunoblot analysis of HER4 phosphorylation in A549 cells after incubation with CM at the indicated concentrations of PM-treated THP1 for 20 min. **g** Immunoblot analysis of HER4 phosphorylation in A549 cells after incubation with CM-Control (Con) at the indicated concentration of rhHBEGF for 20 min. *$P \leq 0.05$, **$P \leq 0.01$, ***$P \leq 0.001$; ns, not significant.

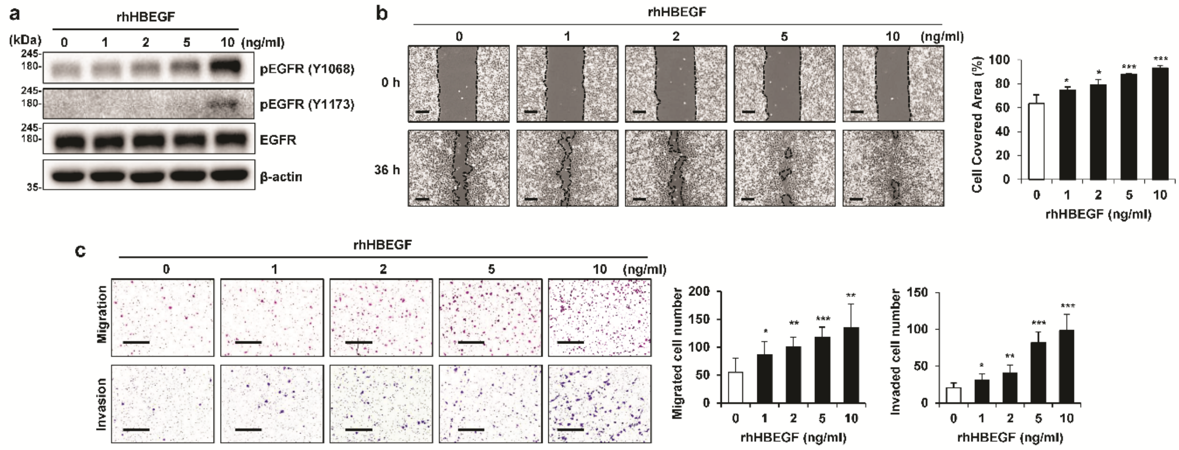

Supplementary Fig. 5 Recombinant HBEGF increases the motility of cancer cells. **a** Immunoblot analysis of EGFR phosphorylation in A549 cells after treatment with the indicated concentration of recombinant human HBEGF (rhHBEGF) for 20 min. β-Actin was used as the loading control. **b** Wound healing assay of A549 cells treated with the indicated concentration of rhHBEGF; images were captured at 0 and 36 h. Scale bar, 250 µm. **c** Transwell migration (top) and invasion (bottom) assays of A549 cells treated with the indicated concentrations of rhHBEGF. Scale bar, 200 µm. *$P \leq 0.05$, **$P \leq 0.01$, ***$P \leq 0.001$; ns, not significant.

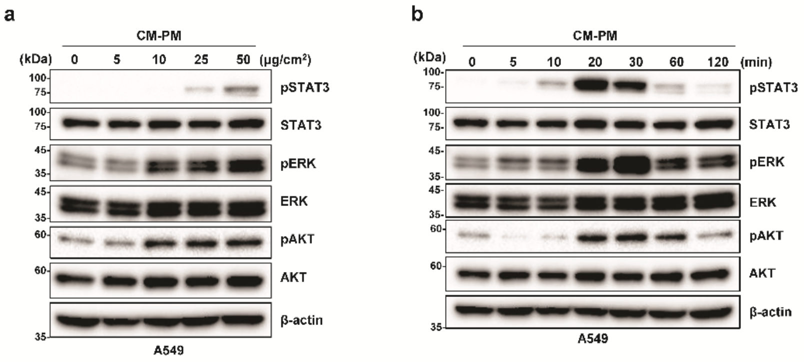

Supplementary Fig. 6 Activation of the EGFR downstream signaling pathway by conditioned media from particulate matter treated cells (CM-PM). **a** Immunoblot analysis of EGFR downstream signaling in A549 cells after treatment with CM-PM for 1 h. β-Actin was used as a loading control. **b** Immunoblot analysis of EGFR downstream signaling in A549 cells after treatment with CM-PM (25 μg/cm^2) for the indicated times.

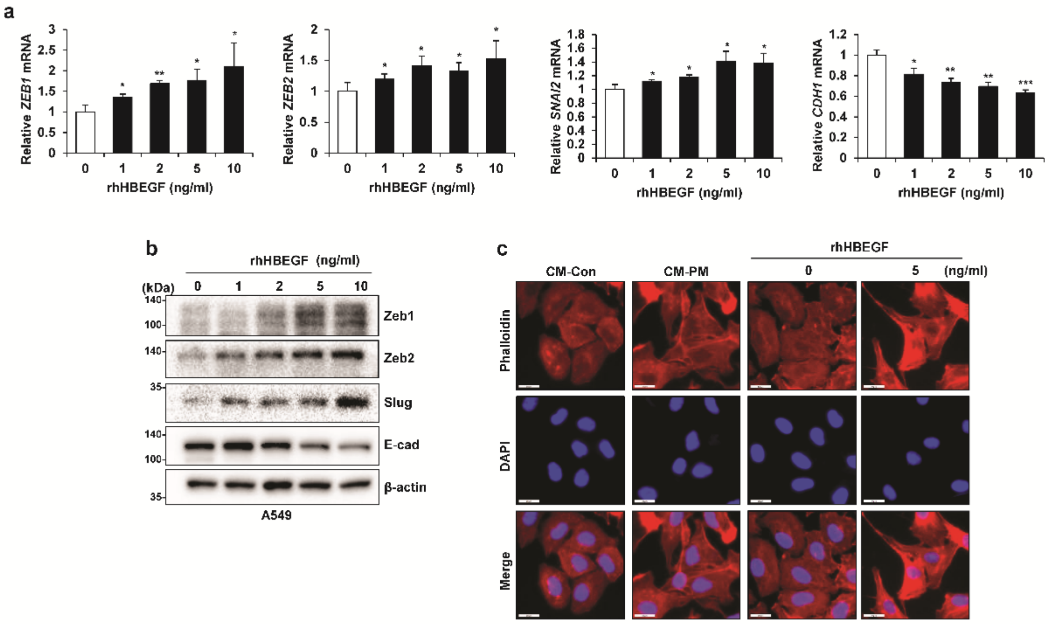

Supplementary Fig. 7 Recombinant HBEGF increases the EMT of cancer cells. **a** qRT-PCR analysis of A549 cells treated with the indicated concentration of rhHBEGF for 24 h. **b** Immunoblot analysis of A549 cells incubated with the indicated concentration of rhHBEGF for 24 h. β-Actin was used as the loading control. **c** Immunofluorescence of A549 cells cultured with the indicated concentration of rhHBEGF for 24 h. Scale bar, 20 μm. $*P \leq 0.05$, $**P \leq 0.01$, $***P \leq 0.001$, ns, not significant.

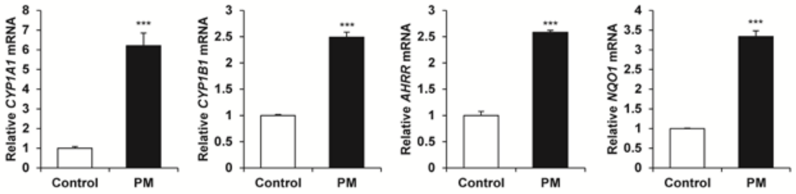

Supplementary Fig. 8 Activation of the AhR pathway in PM2.5-stimulated human cord blood-derived macrophages. qRT-PCR analysis of human cord blood-derived macrophages treated with 25 μg/cm^2 of PM2.5 for 24 h. ***$P \leq 0.001$.

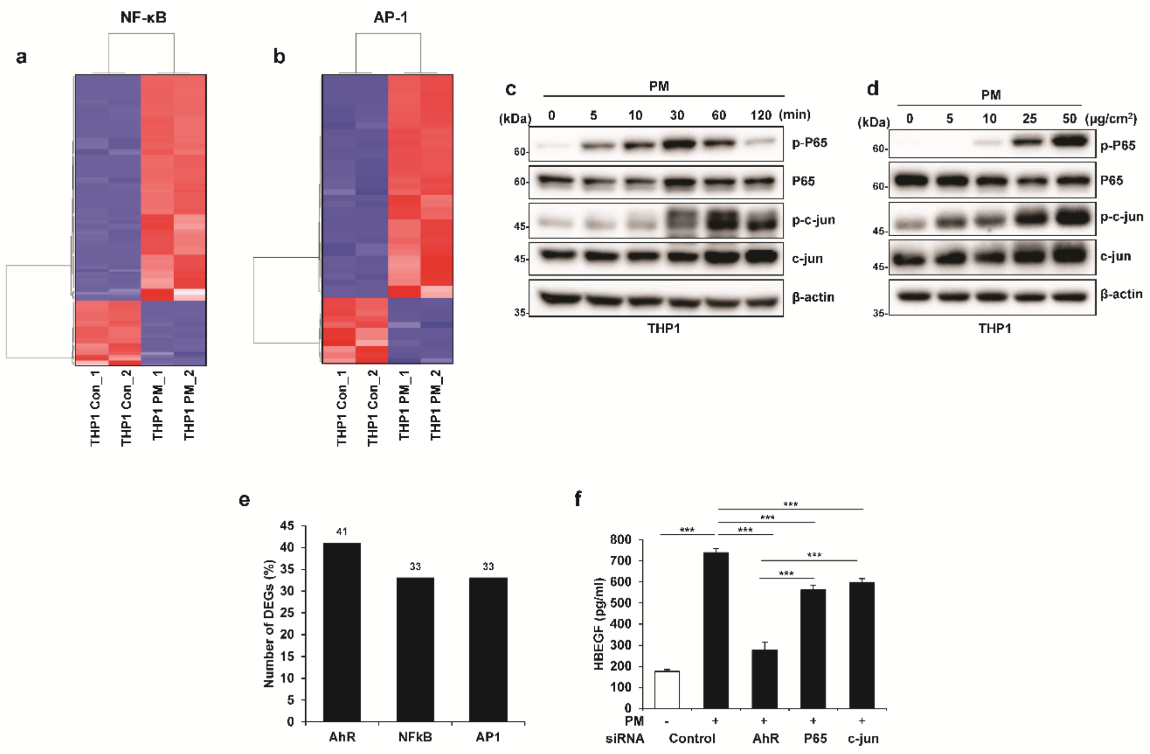

Supplementary Fig. 9 Activation of NF-κB and AP-1 in PM-induced macrophages increases HBEGF expression. **a, b** Heatmap of differential expression of the indicated NF-κB (a) and AP-1 (b) related genes between THP1-Con and THP1-PM cells from RNA-seq datasets. **c** Immunoblot analysis of P65 and c-Jun phosphorylation in A549 cells after treatment with PM (25 μg/cm^2) for the indicated time. β-Actin was used as the loading control. **d** Immunoblot analysis of P65 and c-Jun phosphorylation in A549 cells after treatment with the indicated concentrations of PM for 10 min. **e** Bar graph comparing the number of DEGs from the Target genes in TRRUST database v2. The percentage was calculated from the total number of target genes and the number of DEGs: AhR, 22 and 9; NF-κB, 306 and 102; AP1, 147, and 48, respectively. **f** ELISA of THP1 transfected with 50 nM of siAhR, siP65, and sic-Jun for 24 h and treated with 25 μg/cm^2 of PM for an additional 24 h. *$P \leq 0.05$, **$P \leq 0.01$, ***$P \leq 0.001$, ns, not significant.

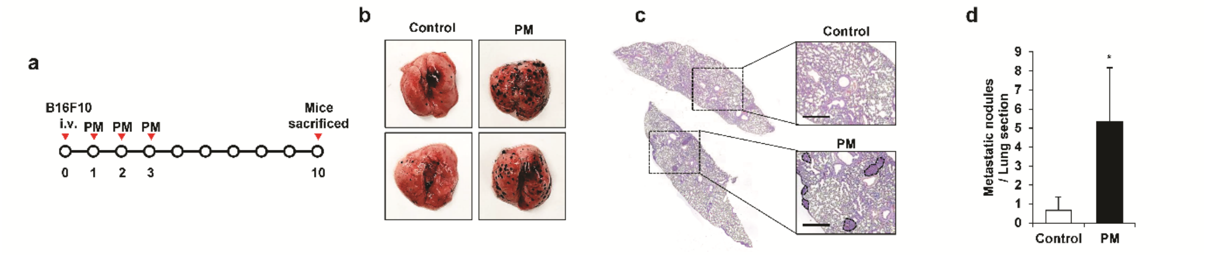

Supplementary Fig. 10 PM increases B16F10 lung metastasis. **a** Schematic illustration of animal study protocol. B16F10 cells were injected through the tail vein, and after 24 h, mice were administered PM through intratracheal injection for 3 days; the mice were euthanized on the tenth day. **b** Representative photos of lungs from PM-treated mice receiving the tail vein injection of B16F10 cells (n = 5 per group). **c** Hematoxylin & eosin (H&E) staining of lung samples harvested from the B16F10 metastasis model. Scale bar, 500 µm. **d** Quantification of average metastatic nodules in the lung sections. *$P \leq 0.05$.

Supplementary Table 1. Primers for quantitative reverse-transcription PCR

Target	Forward Primer (5′→3′)	Reverse Primer (5′→3′)
RPS18	TTCTGGCCAACGGTCTAGACAAC	CCAGTGGTCTTGGTGTGCTGA
SNAI2	TTCAACGCCTCCAAAAAGCC	GATGGGCTGTATGCTCCTG
ZEB1	AGCAGTGAAAGAGAAGGGAATGC	GGTCCTCTTCAGGTGCCTCAG
ZEB2	ATAAGGGAGGGTGGAGTGGA	CGCGTTCCTCCAGTTTTCTT
CDH1	TGAGCACGTGAAGAACAGCA	GCAGAAGTGT CCCTGTTCCA
HBEGF	ACAAGGAGGAGCACGGGAAAA	CGATGACCAGCAGACAGACAG
EGF	TGCAACTGTGTTGTTGGCTACATC	TGGTTGACCCCCATTCTTGAG
EREG	ATCATGTATCCCAGGAGAGTCCAG	GAATCACGGTCAAAGCCACATAT
AREG	AGAGTTGAACAGGTAGTTAAGCCCC	GTCGAAGTTTCTTTCGTTCCTCAG
BTC	TTCACTGTGTGGTGGCAGATGG	ACAGCATGTGCAGACACCGATG
TGFA	AATGACTGCCCAGATTCCCA	GCAGGAACGTACCCAGAATG
NRG1	CACTATACTTCCACAGCCCATC	TGTGCCTACTGTTTTCTACGG
NRG2	CCTGTGCACTGACTGCGCCA	TTACCGGCTGCTGCCTCACA
NRG3	AGCCATGTCCAGCTGCAAAATTAT	GCCGACAAAACTTGACTCCATCAT
NRG4	AACAGATCACGAAGAGCCCTGT	TGGGAATAGTAGGTATCACATAAC
EPGN	TGACAGCACTGACCGAAGAG	CTCATGGTGGAATGCACAAG
CYP1A1	CAAGAGGAGCTAGACACAGTGATT	AGCCTTTCAAACTTGTGTCTCTTGT
CYP1B1	TTCGGCCACTACTCGGAGC	AAGAAGTTGCGCATCATGCTG
AHRR	CAGTTACCTCCGGGTGAAGA	CCAGCGCAAAGCCATTAAGA
NQO1	GACATCACAGGTAAACTGAAGG	GCAGGGGGAACTGGAATATC
